# *In vivo* chronic myocardial infarction characterization by spin locked cardiovascular magnetic resonance

**DOI:** 10.1186/1532-429X-14-37

**Published:** 2012-06-15

**Authors:** Walter RT Witschey, Gerald A Zsido, Kevin Koomalsingh, Norihiro Kondo, Masahito Minakawa, Takashi Shuto, Jeremy R McGarvey, Melissa M Levack, Francisco Contijoch, James J Pilla, Joseph H Gorman, Robert C Gorman

**Affiliations:** 1 Department of Surgery, University of Pennsylvania, Philadelphia, PA, USA; 2 Department of Radiology, University of Pennsylvania, 500 S Ridgeway Ave, Glenolden, PA 19036, USA; 3 Department of Bioengineering, University of Pennsylvania, Philadelphia, PA, USA

## Abstract

**Background:**

Late gadolinium enhanced (LGE) cardiovascular magnetic resonance (CMR) is frequently used to evaluate myocardial viability, estimate total infarct size and transmurality, but is not always straightforward is and contraindicated in patients with renal failure because of the risk of nephrogenic systemic fibrosis. T2- and T1-weighted CMR alone is however relatively insensitive to chronic myocardial infarction (MI) in the absence of a contrast agent. The objective of this manuscript is to explore T1ρ-weighted rotating frame CMR techniques for infarct characterization without contrast agents. We hypothesize that T1ρ CMR accurately measures infarct size in chronic MI on account of a large change in T1ρ relaxation time between scar and myocardium.

**Methods:**

7Yorkshire swine underwent CMR at 8 weeks post-surgical induction of apical or posterolateral myocardial infarction. Late gadolinium enhanced and T1ρ CMR were performed at high resolution to visualize MI. T1ρ-weighted imaging was performed with a B_1_ = 500 Hz spin lock pulse on a 3 T clinical MR scanner. Following sacrifice, the heart was excised and infarct size was calculated by optical planimetry. Infarct size was calculated for all three methods (LGE, T1ρ and planimetry) and statistical analysis was performed. T1ρ relaxation time maps were computed from multiple T1ρ-weighted images at varying spin lock duration.

**Results:**

Mean infarct contrast-to-noise ratio (CNR) in LGE and T1ρ CMR was 2.8 ± 0.1 and 2.7 ± 0.1. The variation in signal intensity of tissues was found to be, in order of decreasing signal intensity, LV blood, fat and edema, infarct and healthy myocardium. Infarct size measured by T1ρ CMR (21.1% ± 1.4%) was not significantly different from LGE CMR (22.2% ± 1.5%) or planimetry (21.1% ± 2.7%; p < 0.05).T1ρ relaxation times were T1ρ_infarct_ = 91.7 ms in the infarct and T1ρ_remote_ = 47.2 ms in the remote myocardium.

**Conclusions:**

T1ρ-weighted imaging using long spin locking pulses enables high discrimination between infarct and myocardium. T1ρ CMR may be useful to visualizing MI without the need for exogenous contrast agents for a wide range of clinical cardiac applications such as to distinguish edema and scar tissue and tissue characterization of myocarditis and ventricular fibrosis.

## Background

The size, location and extent of a myocardial infarction (MI) are important criteria for the evaluation of post-infarction left ventricular (LV) remodeling [1-3]. To detect MI, it is desirable to inject Gd-DTPA as an exogenous, T1 relaxation time shortening contrast agent. The late gadolinium enhanced (LGE) cardiovascular magnetic resonance (CMR) of myocardial infarction has high contrast-to-noise ratio (CNR), discriminating the infarcted region from the neighboring myocardium [4,5]. Despite its high sensitivity, a challenge is the inability to administer paramagnetic contrast agents to patients with severe renal failure, as determined in many hospitals by imaging restrictions based on glomerular filtration rate. Endogeneous contrast agents might be a desirable alternative.

Myocardial tissue relaxation mechanisms, T2 and T1, are generally observed not to be as effective as LGE to discriminate chronic MI characterized by fibrotic scar tissue [6]. The additional relaxation rate contribution from local magnetic fields, water diffusion through magnetic field gradients among other mechanisms, may obfuscate intrinsic T2-weighted contrast mechanisms for detection of MI [7]. In several studies, there was little or no discriminating ability of T2 to detect chronic MI [6]. Alternative endogenous contrast methods based on T1ρ CMR and spin locking have shown some potential in patients to detect acute MI [8,9], but there have been no T1ρ CMR studies of chronic MI in vivo. An additional challenge is the high specific absorption rate of radiation of conventional T1ρ pulse sequences. It is not clear if these methods can be utilized for CMR on clinical MR scanners.

The goal of this study is to characterize chronic MI in vivo using T1ρ CMR. We sought to determine the infarct scar size, mass, transmurality and contrast using T1ρ CMR ina swine model of MI and to correlate these results with LGE CMR and *ex vivo* planimetry*.* T1ρ maps were computed in several animals to determine the feasibility of in vivo T1ρ relaxation time mapping . The potential for T1ρ imaging as a noninvasive, non-contrast imaging modality to detect edema, acute MI, and LV border zone remodeling is discussed.

## Methods

### Animal model of myocardial infarction

A swine MI model analogous to a previously described sheep model was used in a study approved by the Institutional Animal Care and Use Committee (IACUC) at the University of Pennsylvania [10]. The animal was sedated for surgery with ketamine, weighed and transported to the preparatory room. An ear catheter was placed for intravenous access. The animal was positioned and intubated with an endotracheal tube. The surgical sites were shaved and ECG electrodes were applied to the animal’s legs. The animal was transported to the operating room table and general anesthesia was initiated with isoflurane. ECG leads were attached and the animal was secured to the table with soft restraints. Using sterile techniques, venous and arterial sheathes were inserted for fluid administration, intravenous access, intravenous medications and hemodynamic monitoring. The subcutaneous tissue and muscles were divided with a cautery, the 5^th^ rib was resected and the 5^th^ interspace entered. The pericardium was opened and the heart supported in a pericardial cradle.

Nonabsorbable sutures were used to ligate 3–6 coronary arteries, including the left anterior descending artery and D1 and D2 branches, to create a 20–25% area of infarction in the anteroapical MI model (N = 6). 2 nonabsorbable sutures were used to ligate 2 coronary arteries, the left circumflex and proximal posterior descending arteries, to create a 20–25% area of infarction in the posterolateral MI model (N = 1). An intercostal nerve block was created at the surgical site. After an appropriate period of observation, the chest was closed in standard fashion. The skin was closed with a continuous subcuticular closure. The incision was covered with a sealant.

In preparation for imaging experiments at 8 weeks post-infarction, the animal was anesthetized and vascular access was obtained. Following the imaging experiments, the arterial and venous catheters were removed. Adequate homeostasis was ensured. The animal was weaned from anesthesia and moved to the recovery room.

### Magnetic resonance imaging

Dual respiratory and cardiac physiologic gating of CMR pulse sequences was performed by combining information from an intraaortic pressure transducer (Millar Instruments, Inc, Houston Texas) with the signal interfaced to custom physiologic monitoring software in LabView (National Instruments, Inc., Austin Texas).

CMR was performed on a 3 T clinical imaging system (Siemens Healthcare, Erlangen, Germany) equipped with nominal 40 mT/m gradient system, RF body coil transmission, 6 channel body matrix receiver and 6 or 9 (of 18) channel spine RF receiver elements.

#### *T1*ρ *CMR*

Several T1ρ CMR scans were performed in which the spin locking pulse duration (TSL) was varied to perform T1ρ parametric mapping. T1ρ-weighted images were acquired either in late diastoleor mid-systole. A 3D T1ρ-prepared multishot gradient echo (i.e. T1ρ-prepared turboflash) was performed with the following imaging parameters: TE = 1.4 ms, TR = 38.4 ms (4.8 ms per echo), echo train length = 8, resolution = 0.64 mm × 0.64 mm, slice thickness = 2.6 mm, matrix = 192 × 192, flip angle = 25°, 20 slices, spin lock amplitude (*ν*_1_) = 500 Hz, spin lock duration (TSL) = 6, 18, 30, 42, and 48 ms. All spin lock pulses were below FDA-mandated SAR limitations. The T1ρ relaxation time has been found to increase with spin lock amplitude in myocardial tissue [7], therefore it is,in general,desirable to vary the contrast by manipulation of B_1_ subject to SAR constraints. Contrast was evaluated at a single spin lock amplitude in these experiments.

#### Cine CMR

2Dmultislice cardiac cine CMR was performed with a balanced steady-state free precession (bSSFP) imaging sequence with the following imaging parameters: TE = 1.6 ms, TR = 3.2 ms, slice thickness = 4 mm, flip angle = 40°, resolution = 1.28 mm × 1.28 mm, bandwidth = 1184 Hz/pixel, slices = 20–24.

#### Late gadolinium enhanced (LGE) CMR

A 0.1 mmol/kg injection of gadobenatedimeglumine (MultiHance; Bracco Diagnostics, Inc; Princeton, NJ)was delivered intravenously 15 minutes prior to imaging. In one animal, a 0.2 mmol/kg injection was delivered. At 15 minutes a TI scout was performed consisting of a 2D single slice, inversion prepared balanced steady-state free precession (bSSFP) sequence. The TI-Scout was used to determine the optimal time for inversion. The imaging parameters were: slice thickness = 8 mm, TR = 19.5, TE = 1.45, averages = 2, bandwidth per pixel = 965 Hz/pixel, flip angle = 35 degrees, matrix = 192 × 156, FOV = 300 × 243 mm^2^. Following the TI scout, a high resolution 3D, inversion prepared, multishot, spoiled gradient echo (SPGR) acquisition was acquired in late diastole with the following imaging parameters: TR = 591.28 ms, TE = 2.96 ms, averages = 2, matrix = 256 × 256, flip angle = 25 degrees, bandwidth = 399 Hz/pixel, FOV = 350 mm^2^, segments = 16, TI = 200–300 ms. In the animal receiving 0.2 mmol/kg gadobenatedimeglumine, a phase sensitive inversion recovery (PSIR) LGE experiment [11] was performed with the same imaging parameters. LGE imaging slices were positioned to match T1ρ-weightedimages.

### Image segmentation

Two operators (KK and WRTW) performed MI tissue characterization from 3D LGE and T1ρ CMR images and average values are reported (ImageJ, NIMH, NIH, Bethesda, MA). Infarct size was computed by manual segmentation of the endocardial contour and measurement of its perimeter. The percent infarct size was calculated as the ratio of the infarct endocardium perimeter to the total endocardium perimeter. Transmurality was computed from endocardial, nontransmural infarct perimeter and percent transmural infarct calculated as the ratio of the nontransmural to transmural perimeters in LGE and T1ρ-weighted images. Infarct mass was determined by segmentation of ROIs encompassing the entire infarct and the LV volume to the mitral and aortic annular levels. The calculation of tissue mass assumed an average tissue density of 1.06 g/mL. Contrast-to-noise ratio (CNR) was defined as the difference between infarcted and remote myocardial signal intensity normalized by the noise level.

Images were segmented using custom chord segmentation software in Matlab (Natick, MA). Short axis slices were manually traced along the epi- and endocardial surfaces. Each short axis slice was segmented into 20 circumferential wedges and 5 radial sections with respect to the cardiac centroid. Signal intensity measurements were taken from the mid-myocardium (radial sections 2–4) and normalized to the maximum value.Statistical significance was performed by a 2-way ANOVA in Matlab (Natick, MA) at a 5% level of significance.

### Histology

Histology was performed in one animal. Following euthanasia, the complete heart were excised and thoroughly washed in 0.9% normal saline to remove any residual blood. Evans blue and 0.1 M 2, 3, 5-triphenyltetrazolium chloride (TTC) tissue staining was performed. The bilateral coronary ostia were cannulated with 16 gauge angiocatheters and securely snared to prevent backflow. Each coronary was then flushed with 500 ml saline followed by approximately 30 ml 0.5% Evan’s blue in distilled water. The coronary sinus was continuously suctioned during Evans blue instillation to minimize unwanted tissue contamination. After ensuring homogeneous staining of the perfused myocardium, the heart was then sectioned in 5 mm short axis slices from apex to base—being careful to note slice number and orientation. Slices were then incubated for 20 minutes in a phosphate buffered saline solution with 0.1 M TTC. Each slice was completely submerged and turned every five minutes to guarantee full coverage. Following incubation, all slices were photographed in natural light using a commercially available digital camera. Once complete, Evans blue staining demarcates perfused tissue while TTC staining highlights viable myocardium. Tissue that is blue with no evidence of TTC staining denotes scar that has undergone angiogenesis and developed collateral flow during extracellular matrix remodeling.

## Results

### Infarct, blood and myocardium T1ρ contrast

**Figure 1:**
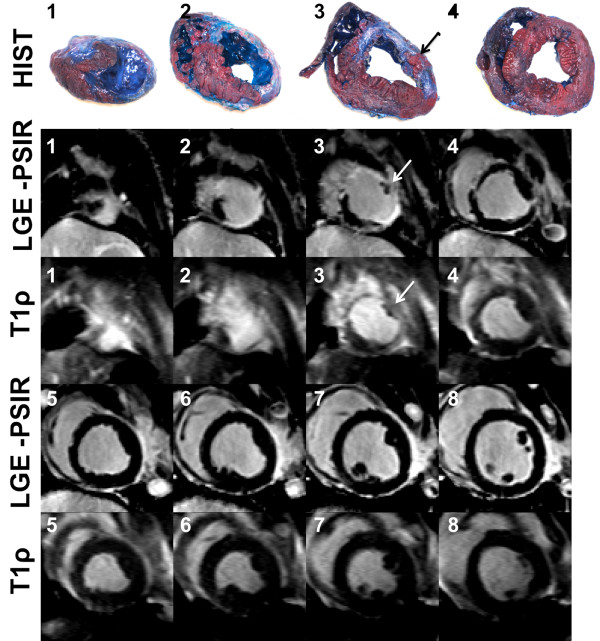
**Anteroapical MI at 4 weeks post-infarction. Matched histology, LGE-PSIR and T1**ρ **(B**_**1**_ **= 500 Hz, TSL = 50 ms) images are labeled according to slice number from LV apex to base.** Fibrotic scar tissue appears as an area of enhanced signal intensity on both LGE-PSIR and T1ρ-weighted images in slices 1–5.T1ρ can visualize small features such as the posterior papillary muscle (black and white arrows in slice 3), which appears non-fibrotic amidst a larger section of fibrotic myocardial tissue using all three methods.

**Figure 2:**
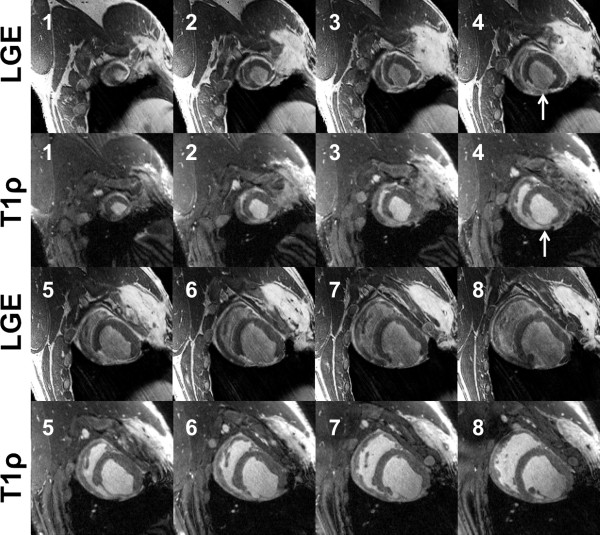
**Posterolateral MI at 4 weeks post-infarction. Matched LGE and T1**ρ **(B**_**1**_ **= 500 Hz, TSL = 50 ms) images are labeled according to slice number from LV apex to base.** The position of the posterolateral scar is indicated in slice 4. The scar appears extends from the apex to the mitral valve level on the posterolateral wall.

**Figure 3:**
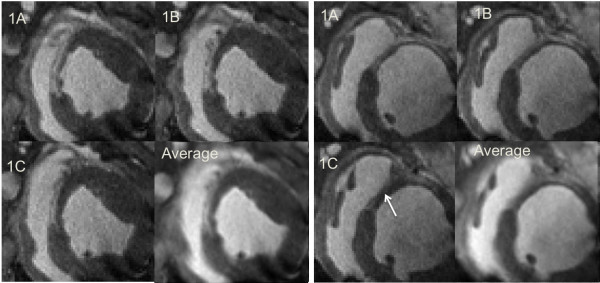
**The appearance of partial volume effects on T1**ρ**-weighted images over an area containing regional infarct transmurality.** Infarct size can be underdetermined as shown for the situation on the left. The composite (average) slice suggests that the infarct is transmural. This is only true, however, only for the most apical slices (A,D), whereas the more basal slices (B,E) and (C,F) are non-transmural. The situation on the right demonstrates that the composite slice can have a heterogeneous appearance of varying transmural signal intensity, whereas thinner slices show that this is not the case. Instead, the infarct has a discrete appearance (i.e. all-or-none, infarct or myocardium). In most cases, the contrast between the LV blood and the infarct can be improved using thinner slices.

T1ρ-weighted images from two exemplary studies eight weeks post-MI illustrate the achievable in vivo contrast in anteroapical (Figure1) and posterolateral MI (Figure2). Long spin lock durations (TSL > 40 ms) yield the highest contrast between myocardium and infarct tissue. Ventricular blood and edema in the intercostal space have the highest signal intensity on account of the longest protonrelaxation times. The healthy myocardium has suppressed signal, whereas the infarct appears bright because of the relatively longer relaxation time compared to the healthy myocardium. The contrast-to-noise ratio (CNR) in LGE and T1ρ CMR was 2.8 ± 0.1 and 2.7 ± 0.1 (Figure3). CNR was not computed for LGE-PSIR images (Figure1) because of the low sample size (N = 1). In one animal, histology confirmed that the infarct tissue at 4 weeks had undergone fibrosis, development of angiogenesis and collateral flow. The histological appearance of the infarction was similar to LGE and T1ρ CMR scans.

Compared to LGE CMR, the ventricular blood in T1ρ CMR contrast appears much brighter because blood in the LV chamber has the longest relaxation time. This may be a source of error at the endocardial infarct boundary. The surrounding blood pool reduces the conspicuity of the infarct at the septal wall and may be eliminated in the future by suppression of flowing blood by incorporating a preparation RF pulse to suppress flowing magnetization [12].

### Regional infarct transmurality and partial volume effects

All animals exhibited transmural infarcts, although at the borderzone T1ρ imaging was able to delineate regions of infarcts occupying only part of the wall thickness. Non-transmural infarct comprised 6% of the total infarct volume in these animals and 1.3% of the total LV volume.

**Figure 4:**
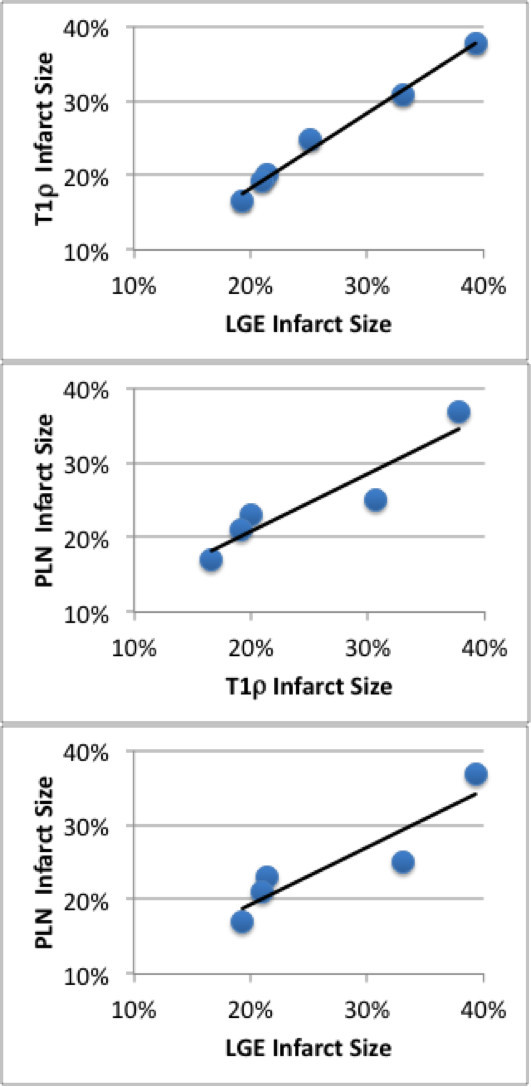
**Correlation between infarct size measured by T1ρand LGE CMR and planimetry.** High correlation was measured infarct size (T1ρ/LGE R^2^ = 0.99, T1ρ/PLN R^2^ = 0.87, LGE/PLN R^2^ = 0.82).

Voxels containing mixtures of blood, myocardium or infarct tissue reduce conspicuity of the boundary, the ability to detect infarct size or the appearance of a transmural infarction at its boundary with the myocardium. Partial volume effects are important to consider because of the potentially increased slice thickness in clinical scans, which may compromise spatial resolution for scan time. These effects are shown in Figure4.

### Infarct size and mass

**Figure 5:**
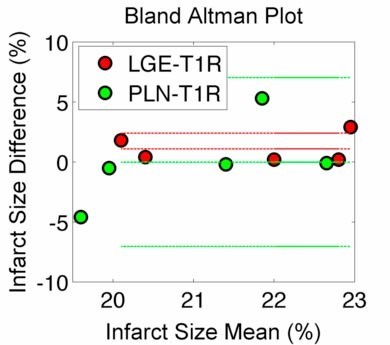
**A Bland-Altman diagram showing the difference in measured size using LGE and T1ρ(red) CMR and planimetry and T1ρCMR (green).** T1ρ was not significantly different from either LGE or planimetric measurement of infarct size.

Infarct size (% endocardial surface) measured by T1ρ CMR (21.1% ± 1.4%) was not significantly different from LGE CMR (22.2% ± 1.5%) or planimetry (21.1% ± 2.7%; p < 0.05) and there was high correlation between T1ρ and LGE infarct size (Figure4). The dual Bland-Altman plots comparing LGE-T1ρ and planimetry (PLN)-T1ρ **(**Figure5) suggest that there is negligible difference in the infarct size using the different measurement methods, although a greater variation in the created infarct size may further validate the correlation between measurements. Mean anteroapicalinfarct mass was 10.9 g or 10.0% of the mean LV mass 108.6 g.

### T1ρ relaxation time mapping

**Figure 6:**
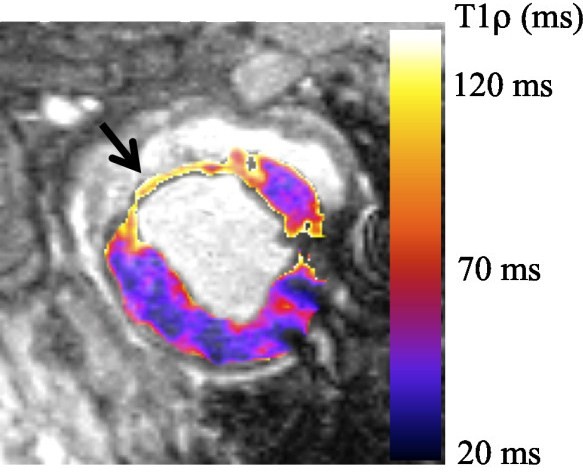
**T1ρrelaxation time map from a single animal 8 weeks post-MI.** The infarct ^1^ H nuclear relaxation time in vivo are measured T1ρ_infact_ = 91.7 ms and T1ρ_myocardium_ = 47.2 ms. Relaxation time are twice as long in the myocardial infarction than the myocardium, which means that T1ρ-weighted images have high signal intensity at the infarct and low signal intensity in the myocardium

T1ρ relaxation time maps from a single animal 8 weeks post-MI with a posterolateral MI are shown in Figure6. T1ρ relaxation times were significantly different in the remote, borderzone and infarct regions (p < 0.01). The T1ρ relaxation time was nearly two times longer in the infarct than in the remote myocardium (T1ρ_infarct_ = 91.7 ms, T1ρ_remote_ = 47.2). The borderzonerelaxation time wasintermediate both relaxation times (T1ρ_borderzone_ = 47.2 ms) suggesting that these voxels contain a mixture of scar and remote myocardial tissues. The large difference in relaxation times (ΔT1ρ = 44.6 ms) allows for T1ρ-weighted images obtained at intermediate spin lock periods to obtain high contrast between scar tissue and remote myocardium.

## Discussion

We characterized myocardial scar tissue, remote myocardium and blood contrast-to-noise ratio, total infarct size with T1ρ-weighted imaging and T1ρ relaxation times in several animals in vivo. Preliminary results of this study were presented for a subset of the animals studied here [13]. These studies demonstrate that T1ρ CMR has the potential to noninvasively characterize chronic MI size, location and regional wall scar transmurality without exogenous contrast agents.

The primary observation was that measurement of infarct size using T1ρ CMR was not significantly different from LGE and both tended to overestimate infarct size compared to planimetry. Differences in underlying contrast mechanisms between T1ρ and LGE CMR suggest that there might be differences in measured or apparent infarct size, however this was not observed experimentally. Nevertheless, the observation of infarct size equivalence finds T1ρ to be a highly potential candidate for an endogenous contrast CMR method. These results suggest that T1ρ CMR may be useful for a wide range of clinical applications in which myocardial tissue characterization is essential, including myocarditis, fibrosis secondary to hypertrophy, as well as acute and chronic scars. We anticipate, however, T1ρ methods will augment rather than replace LGECMR. Although there was no significant difference in infarct size, there are several potential advantages that T1ρ may have compared to paramagnetic contrast agents. T1ρ contrast is a result of nuclear interactions that occur on a low frequency motional timescale on the order of the applied RF field *ω= γВ*_1_. There is the potential to sensitize the MR contrast to different water proton interactions in tissue, including chemical exchange, proton dipole-dipole coupling, diffusion, among other low frequency mechanisms by varying the RF amplitude. These contrast mechanisms have been useful for imaging a wide range of pathologies, including cartilage degeneration in osteoarthritis, degeneration of the intervertebral disc, and monitoring tumor growth and response to treatment. While the continuum of spin lock amplitudes provides access to a range of contrast, improved contrast to distinguish infarct and remote myocardium was found to increase with spin lock amplitude [7].

It is important to compare the theoretical performance of the relaxivity of paramagnetic and endogenous contrast agents and their effect on longitudinal (R1) and rotating frame (R1ρ) relaxation rates. The water ^1^ H longitudinal relaxation rate, 1/T1,at 3 T is affected by both the chemical and pharmacologic properties of low molecular weight paramagnetic contrast agents. Paramagnetic relaxation enhancement is believed to have two contributions: [1] relaxation enhancement of a water molecule directly coordinated to the gadolinium ion, which is dominated by the rotational correlation time of the Gd (III) complex and [2] relaxation enhancement of water ^1^ H through dipolar coupling and translational diffusion nearthe gadolinium complex [14,15]. Gd-chelates also function as drugsand distribute nonspecifically throughout the plasma and interstitium. The pharmacokinetic properties of gadolinium uptake into fibrotic myocardium result in a larger relative quantity of contrast agent in the extracellular space of fibrotic tissue characterized by prolonged wash-in and wash-out kinetics [4].

It has been challenging to determine the origin of the water proton rotating frame relaxation, T1ρ,because there is not known to be a single dominant relaxation mechanism in vivo. The rotating frame relaxation rate, 1/T1ρ, has been studied in protein solutions and was found to depend on the molecular weight, concentration, protein structure and solution pH [16,17]. The latterindicatesan important contribution of chemical exchange to the relaxation rate. Other possible contributions include dipolar-coupling between water protons bound to the protein through hydrogen bonding and water proton magnetization transfer. It is hypothesized from T1ρdispersion experiments performed on infarcted and non-infarcted myocardial tissue explants thatT1ρ differentiates water protonsbecause of observed increased water protonmobility in scar [7]. Increased water mobility leads to shorter nuclear rotational correlation times and longer transverse relaxation time. Increased water mobility in collagenous scar is not a new finding and would agree with previous observations of increased water proton diffusivity in diffusion-weighted CMR [18,19].

T1ρ CMR may overcome several challenges to accurate measurement of infarct size by LGE. Noncontrast methods are very important for patients who have severe renal disease. There are reported discrepancies regarding the accuracy of LGE CMR. It has been suggested that LGE measurement of infarct size may be inaccurate if there are regional changes in contrast kinetics [20] or changes in the timing protocol between injection and image acquisition. In other studies LGE is reported to be highly accurate over a range of delay times and show excellent correspondence between infarct size and pathological staining [21]. The experiments performed here also suggest that LGE reliably determines infarct size in chronic MI.

T2-weighted images showsignal enhancement in acute MI [12,22,23], correlating with an increase water content and edema [24,25]. T2-weighted CMR is ambiguously sensitive to chronic MI, with no observed increase in signal intensity or T2 relaxation at the site of infarct [6] or changes in T2 that were dependent on the location of infarction [26]. These mixed results would reinforce the hypothesis that there are low frequency relaxation mechanisms unrelated to disease that obscure contrast [7]. T2-weighted CMR has been observed to overestimate the size of MI compared to LGE in acute MI [22]. This has prompted further research to understand T2-weighted enhancement as an area at risk rather than the infarct scar size alone [27]. Therefore a measurable difference between T2-weighted and LGE would enable the evaluation of interventional procedures to reduce infarct size from the initial area at risk. Further investigation is needed into T1ρ contrast in acute, stunned or hibernating, myocardium and the relationship to collagen type and infarct maturation. Compared to endogenous contrast mechanisms based on T2 relaxation, T1ρ overcomes low-frequency relaxation mechanisms that reduce scar and remote myocardium contrast-to-noise ratio. The spin locking pulse increases the myocardium-scar tissue contrast because ΔT1ρ is greater than ΔT2, so that at the optimal evolution period (TSL or TE), the contrast-to-noise ratio was observed to improve*ex vivo*[7].

Magnetization transfer contrast or magnetization transfer ratio imaging methods are typically different from T1ρ imaging methods by virtue of their off-resonance RF pulse preparation, which is designed to further suppress signals from water protons undergoing exchange with a broad macromolecular pool. The myocardium has high macromolecular content and so MT techniques are useful to facilitate contrast between infarct and blood, the latter containing little or no MT effects [28]. In pathologic cardiac tissue, these methods are reported to distinguish normal and stunned myocardium, which is unlike LGE CMR [29]. Off-resonance saturation also has the potential to be highly specific to high concentration metabolites in the mM range (e.g. creatine) through the chemical exchange saturation transfer (CEST) effect. These methods have the advantage over on-resonance spin locking or magnetization transfer contrast because they may be highly specific to a single metabolite [30].

There are some challenges to T1ρ CMR in vivo. In some images a region of low signal intensity was measured on the endocardial surface surrounding the heart, but appearing most prominently at the infarct-blood surface. It’s not clear whether this is an artifact associated with limited spatial resolution and Fourier imaging (Gibbs phenomenon) or is related to motion throughout the spin lock and acquisition. The T1ρ pulse sequence is sensitive to magnetic field heterogeneity. Image artifacts can be a result of nutation of the magnetization around the effective field in the rotating frame [31]. These artifacts appear qualitatively similar to dark signal intensity bands encountered in balanced steady-state free precession pulse sequences [32].

Achallenge to T1ρ CMR isthe high specific absorption rate of radiation (SAR) delivered to subjects. The high SAR limits the maximum RF power and duration used for spin locking and therefore limits contrast. We addressed this problem by placing limits on the duration and amplitude of the spin lock pulse. Future study to maximize contrast in the context of these limitations is important. Typical limitations encountered were a maximum B_1_ = 500 Hz, for continuous wave irradiation for 50 ms and TR = 3 s. It should be understood that these limitations are hardware specific (e.g. dependent on powerloss in the RF amplifier and transmit coil, design of the transmit coil) and dependent on the vendor bioelectromagnetic model of the subject used to estimate tissue heating. This model may not be appropriate for swine. All animal experiments were performed with the vendor-supplied SAR monitor enabled. Another challenges is the spatiotemporally varyingmagnetic field heterogeneityat the posterolateralmyocardium and physiologic motion of the heart and lung. The temporalmagnetic field heterogeneitywas mitigated by dual cardiac and respiratory physiologic gating.

## Conclusion

T1ρ CMR accurately measures the size of chronic MI and is highly correlated with measurements by late gadolinium enhancement CMR and planimetry. There is the potential for T1ρ to be useful method to generate endogenous contrast as an alternative to in vivo LGE in sequential, follow-up studies or in patients unable to receive contrast.

## Competing interest

'The author(s) declare that they have no competing interests'.

## Author’s contribution

WRTW: study design, analysis and interpretation of data, statistics, drafting of the manuscript. GAZ: analysis and interpretation of data, revising the manuscript critically for important intellectual content. KK: study design, analysis and interpretation of data, statistics, revising the manuscript critically for important intellectual content. NK: study design, analysis and interpretation of data, revising the manuscript critically for important intellectual content. MM: study design, analysis and interpretation of data, revising the manuscript critically for important intellectual content. TS: study design, analysis and interpretation of data, revising the manuscript critically for important intellectual content. JRM: study design, analysis and interpretation of data, statistics, revising the manuscript critically for important intellectual content. MML: analysis and interpretation of data, revising the manuscript critically for important intellectual content FC: analysis and interpretation of data, revising the manuscript critically for important intellectual content. JJP: study design, analysis and interpretation of data, statistics, revising the manuscript critically for important intellectual content. JHG: study design, analysis and interpretation of data, statistics, revising the manuscript critically for important intellectual content. RCG: study design, analysis and interpretation of data, statistics, revising the manuscript critically for important intellectual content. All authors read and approved the final manuscript.
